# Detecting the interaction between urban elements evolution with population dynamics model

**DOI:** 10.1038/s41598-023-38979-w

**Published:** 2023-07-31

**Authors:** Min Jin, Lizhe Wang, Fudong Ge, Jining Yan

**Affiliations:** 1 School of Computer Science, China University of Geosciences, Wuhan, 430074 China; 2 Hubei Key Laboratory of Intelligent Geo-Information Processing, China University of Geosciences, Wuhan, 430074 China

**Keywords:** Ecosystem ecology, Population dynamics, Sustainability

## Abstract

Exploring the evolution of urban elements can improve understanding of the developmental process of city and drive such development into a better direction. However, the non-linearity and complexity of changes in urban elements have brought great challenges to understanding this process. In this paper, we propose a cross-diffusion partial differential equation based on ecological dynamics to simulate the evolutionary process of urban elements from the microscopic viewpoint. The interaction between urban elements is simulated by constructing a non-linear and spatiotemporal change equation, and the main influence between elements is evaluated by the key parameters in the discussed equation. Our model is first experimented to time-series data on population density and housing prices to analyzes the interaction of these two elements in the evolution process. We then extend the model to label data, land cover data, to obtain a quantitative expression of the interaction between different land types in the process of urban land cover change.

## Introduction

Researches on the development of urban elements have been a fascinating field in recent decades^1,2^. Cities play a crucial role in accommodating a significant proportion of the global populace, and this trend is expected to persist and intensify in the foreseeable future^3,4^. The burgeoning interest in this domain can be attributed, in part, to the intricate and multifaceted nature of urban phenomena, which continues to pose a myriad of unresolved questions and knowledge gaps that warrant further investigation and inquiry^5^. An overarching objective in the study of urbanization is to enhance our understanding of city development processes and utilize this knowledge to facilitate a shift towards a more sustainable path of urban development^6,7^.

With the efforts of generations of outstanding scientific researchers, a number of impactful works have been witnessed on dealing with the development of urban elements. Research on the relationship between urban elements can be divided into two categories. One is concerned with filtering out those factors that play a leading role in changing a specific event when there are many factors at play, analyzing the driving force, and simulating the driving process based on the leading factors^8,9^. A typical representative of this type of research is the analysis of the drivers of the land-use change process^10,11^. Another category seeks to model the relationship between factors directly, without weeding out the dominant factors, and to analyze the evolution of elements based on the results obtained. A typical representative of this type of research is the analysis of the interaction between elements of panel data^12^.

The main process of interest for the research method that focuses on driving forces is land-use change, as the core objective is to first identify the driving factors that play a leading role in land-use change. Typical used methods include the logistic regression^13^, the analytic hierarchy process (AHP)^14^, and the principal components analysis (PCA)^15^, etc. Based on the identification of the dominant driving factors, the driving force is further investigated, and the driving process is simulated. In this stage, commonly used models include cellular automata (CA)^16^ and a number of improved algorithms that have been extended to CA^17,18^. For example, using geographically weighted regression (GWR) causes CA’s transformation rules to be spatially non-stationary^19^. The combination of the agent-based modeling (ABM) model and the CA model reflects the multi-agent impact process of human activities on land-use change^20^. In addition, machine learning methods have been used to improve the uncertainty generated by artificially setting rules in the CA model^21^ and the improved FLUS model, which has been combined with artificial neural network (ANN), system dynamic (SD), and CA models^22^. Another type of algorithm is the CLUE-S model^23^, which includes the non-required space module, the space allocation module, and the extension of this model. The Markov, the SD, and the random forest algorithm are used to calculate the total land requirements of the non-spatial modules in the CLUE-S model.

When examining the element relationship with panel data, surveys can be classified according to whether a statistical method is used. In the statistical relationship-based model, scientists usually pre-examine some factors and use statistical analysis methods, such as correlation analysis or regression analysis, to examine the relationship between these factors. Ultimately, a statistical model was used to express the relationship between the factors. Models that do not involve spatial heterogeneity typically use linear regression models, such as piecewise linear regression^24^, among other options. With an emphasis on the spatial relationship between elements, a number of spatial econometric models have emerged. The first to be proposed was the spatial autoregressive (SAR) model^25^. The SAR is the modeling of Tobler’s first geographic law, which describes spatial autocorrelation. Some of the more commonly used spatial econometric models include the spatial lag model (SLM, also known as the SAR), the spatial error model (SEM)^26^, and the spatial Durbin model (SDM)^27^. The SDM is a combined and augmented form of the SLM and the SEM that is constructed by adding appropriate constraints.

Besides being involved with the land-use drivers, the GWR model also can be used to study factor relationships based on panel data^28^. GWR illuminates the spatial variation and associated driving factors of the research object at a given scale by establishing a local regression equation at each point in the spatial domain that can be used to predict future outcomes. It has the advantage of better performance, since it takes into account the local effects of spatial objects. And thus derived semiparametric geographically weighted regression (SGWR)^29^, Fast geographically weighted regression (Fast GWR)^30^. Wang et al. also proposed a statistical method called GeoDetector^31^. The core idea of GeoDetector is based on the assumption that if an independent variable has a significant impact on a dependent variable, then the spatial distributions of the independent and dependent variables should be similar.

Among these methodological models, scholars have invariably focused on the spatial heterogeneity between time-series data items. Describing the spatial heterogeneity of elements in the process of time change has become of special interest to scientists. In addition to these models, one particular group of scientists is trying to understand the urban development process from a different angle. For example, researchers have carefully explored the laws and models of urban development, including some physicists who have made brilliant contributions^32,33^. Specifically, these researchers model city growth from a physical perspective by applying models from typical research areas of physics to questions of city growth and urban development^34,35^. Among them, Juste Raimbault et al. used the reaction–diffusion equation to study the growth of urban population. Three parameters, namely aggregation, diffusion and growth speed parameters, were used to reflect the change rule of urban population density, which reflected the effectiveness of the simple model^36,37^. In addition to the reaction–diffusion model, a gravity-based model and correlated percolation have also been considered in the exploration of the law of urban development^38,39^. These models can provide valuable perspectives on urban development theories and generate models that can be used to predict urban growth.

Combining the various models of the development process of urban elements that have been advanced by researchers^40^, in this article, we adopt the cross-diffusion model in pattern dynamics to simulate the mutual evolution process between different elements. The self-diffusion element indicates the spatial overflow or propagation process of the elements, and the cross-diffusion element characterises the interaction between the elements. At the same time, since the model was created for each spatial grid, it exhibits spatial heterogeneity.

In this paper, we first explored the development process of urban elements from the perspective of reaction–diffusion equation. We extended a well-known model from the field of chemistry and biology to the development of urban systems. Secondly, since the parameters of our model could be identified from the actual data, the model provided an explicit expression of the interaction between urban elements. Finally, considering different data types, we applied the model to three kinds of data: population density, housing price and land cover, and studied the correlation between these three kinds of data.

The remain of this paper is divided into five sections. We first present our experimental results in “Results” section, and then comes our “Discussion” section. Next, we illustrate the setup and composition of our algorithmic model in “Methods” section. Finally, we introduce our research area and data processing in “Materials” section.

## Results

In the experimental part, we first feed population density data and house price data into our model to obtain the evolutionary relationship between population density and house prices, and then analyze the results to complete the simulation of unlabeled data. In the next step, we perform experiments for different types of land cover data. The process of the experiment consist of treating each land cover type as an element, performing a mutual evolution simulation, and completing the simulation of the label data.

### Poplation density data and house price data

**Figure 1:**
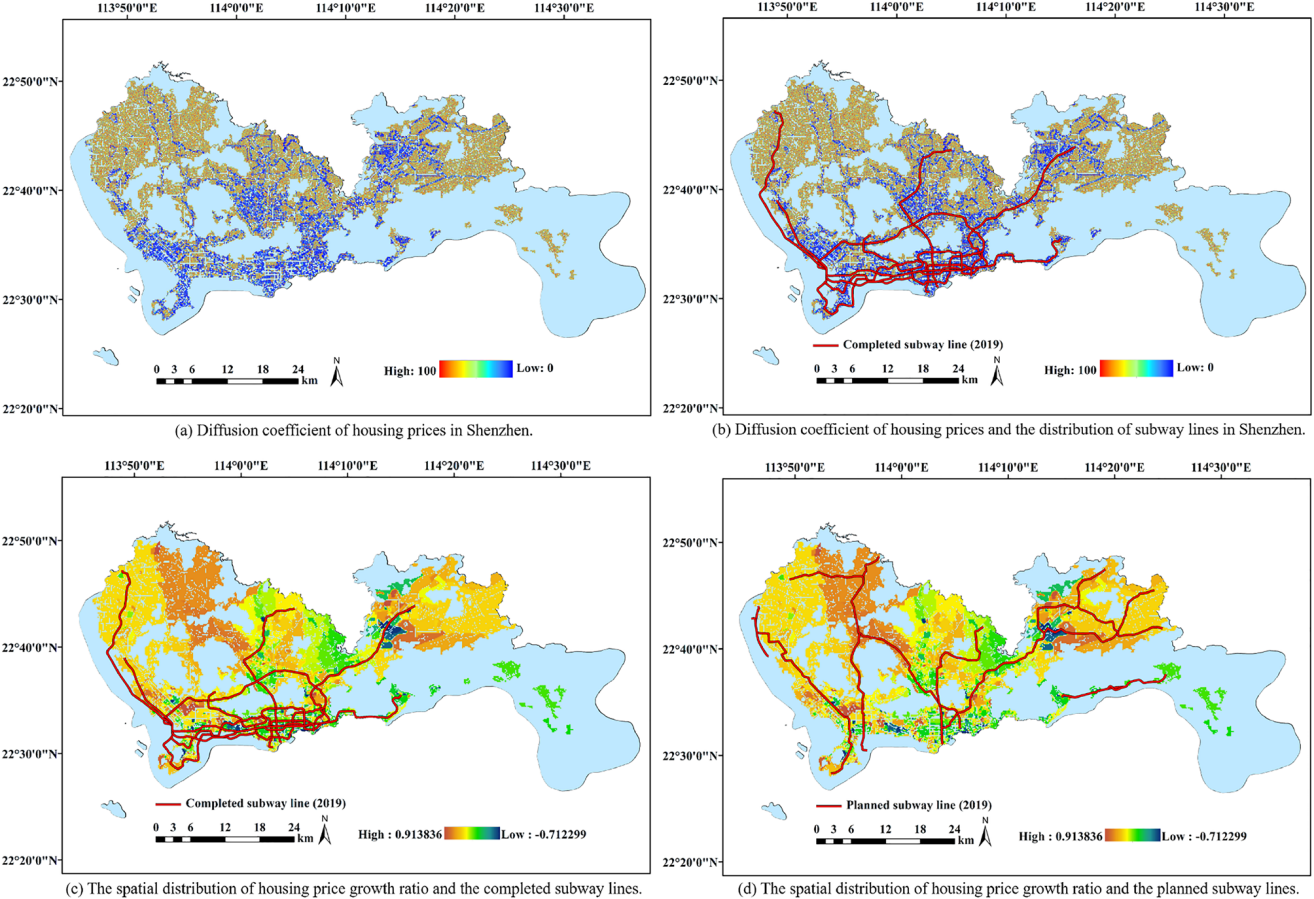
The correlation between the diffusion coefficient of house price and subway lines. The figure was created by QGIS (QGIS Desktop 3.22.4, https://www.qgis.org).

Based on house price data, and considering the impact of population density changes on house price growth, we obtain the following data results. Figure 1a parades the spatial distribution of the diffusion coefficient. It can be seen that the diffusion coefficient of housing prices in Shenzhen exhibits low distribution in the south, high distribution in the north, high in the east, and low in the west, which means that in the north and east of Shenzhen, the higher house price range radiating outward is wider. We compare these findings with the subway lines in Shenzhen, as demonstrated in Fig. 1b. It is found that the diffusion coefficient of housing prices in Shenzhen basically increased outward along subway lines. At the same time, the diffusion coefficient of housing prices in areas close to subway lines is low, which also means that housing prices in Shenzhen radiated outward along subway lines. This also can be seen in Fig. 1c, the proportion of housing price growth ratio in Shenzhen from 2011 to 2019. At the same time, based on the housing price in 2011, along the subway lines that have been built in Shenzhen in 2019, it can be seen that the growth rate of house prices in Shenzhen is generally low. As can be seen in Fig. 1d, along the subway lines planned to be built in Shenzhen after 2019, the increase in housing prices is even greater. With the completion of the subway, the housing prices in this area gradually spread outward and reached a higher price, so the diffusion coefficient also decrease.

**Figure 2:**
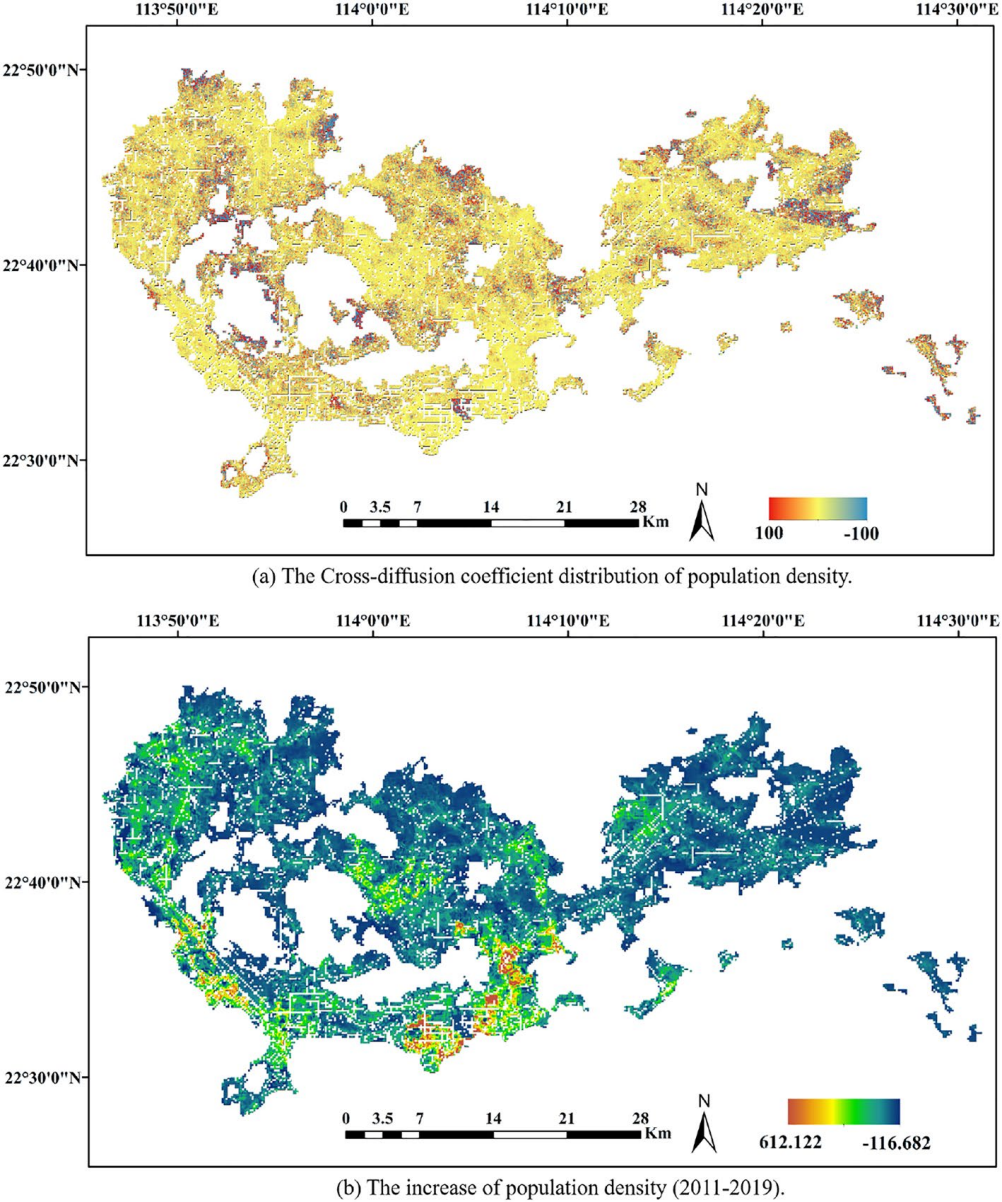
The correlation between diffusion coefficient and subway lines. The figure was created by QGIS (QGIS Desktop 3.22.4, https://www.qgis.org).

From the result of Fig. 2a, the cross-diffusion coefficient of the corresponding population density is distributed. The increase in population density is exposed in Fig. 2b. Overall, the distribution of the cross-diffusion coefficients is consistent with the increase in population density. In regions where the cross-diffusion coefficient is found to be unstable, negative population growth occurred due to the properties of the diffusion equation. At the same time, a positive cross-diffusion coefficient also means that the housing prices in this area are increasing toward the area with population growth. A negative cross-diffusion coefficient indicated that housing prices are increasing from areas with lower populations to areas with higher populations. This characteristic, reflecte by the cross-diffusion coefficient in the diffusion equation, is consistent with the actual situation.

**Figure 3:**
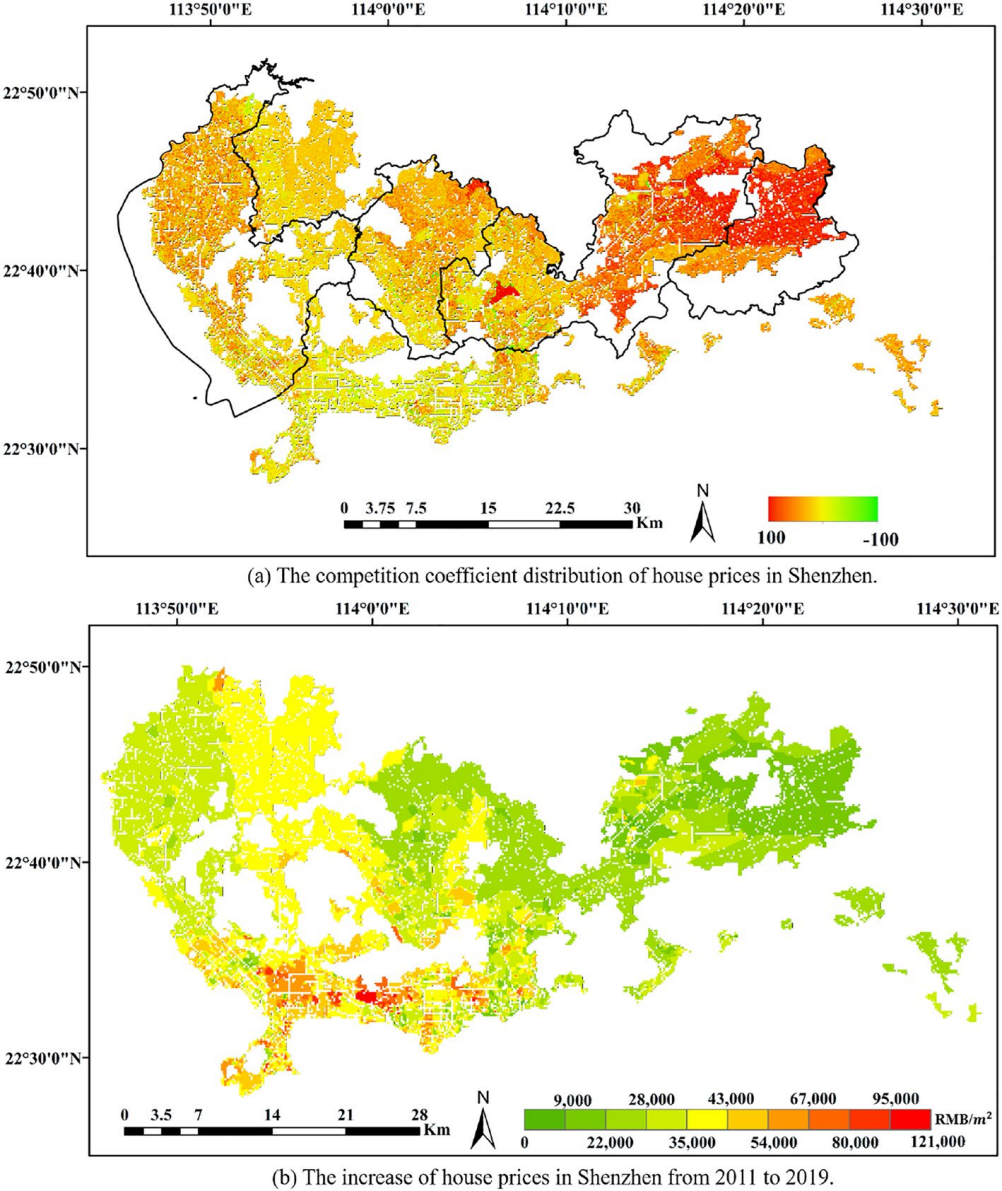
The correlation between competition coefficient and increase in house prices (RMB/$m^2$). The figure was created by QGIS (QGIS Desktop 3.22.4, https://www.qgis.org).

From the spatial distribution of the competition coefficient, it can be found that, as displayed in Fig. 3a, the internal competition of housing prices is also more intense in areas with larger growth coefficients. At the same time, compared with the overall growth rate of house prices, on view of Fig. 3b, the competition within the region with more house price appreciation is more intense. This means that in these areas, the self-growth potential of housing prices is still relatively large. At the same time, in these areas, the phenomenon of housing price competition between sub-regions, that is, real estate speculation, may be more serious.

### Land cover data

In the simulation experiment for land cover data, we first conducte a preliminary analysis of the land cover change in Shenzhen, as shown in Table 1 below. From the figure, we can see that Baoan District had the most newly-added impervious areas, and Yantian District had the least. From the perspective of the proportion of new impervious areas, Guangming District and Dapeng District have the highest and lowest proportions of new areas, respectively. Therefore, we select those four districts for the simulation experiments.

**Table 1:** The changes of impervious areas in various districts of Shenzhen.

District	Baoan	Dapeng	Futian	Guangming	Longgang	Longhua	Luohu	Nanshan	Pingshan	Yantian
New area ($\text {km}^2$)	16.18	1.65	1.09	8.69	11.65	8.42	0.76	4.13	6.97	0.54
New area proportion (%)	4.17	0.53	1.36	5.12	2.76	4.38	0.89	2.57	3.86	0.74

#### The experiment in Baoan District

**Figure 4:**
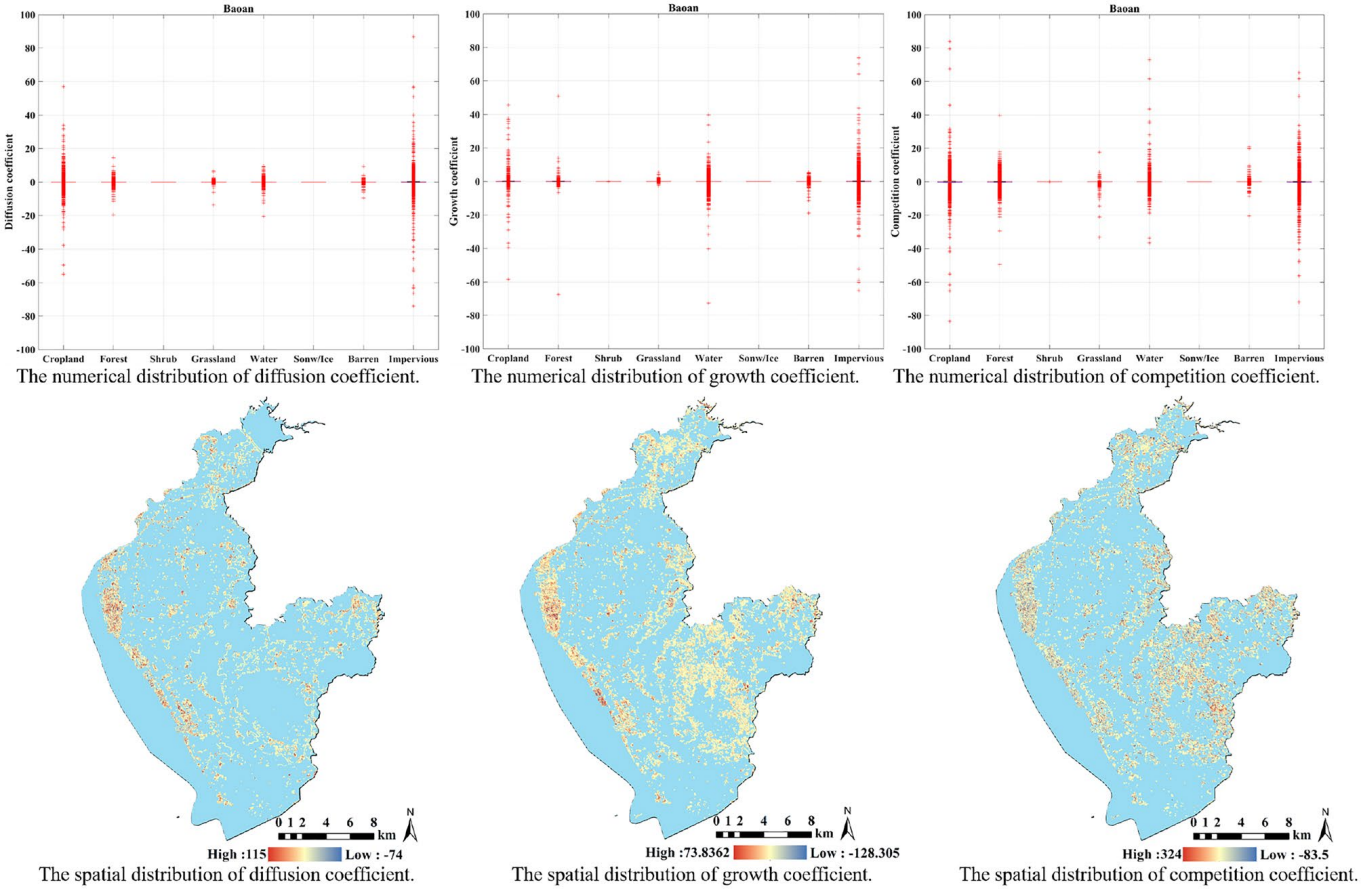
The distribution of different coefficients in Baoan. The figure was created by QGIS (QGIS Desktop 3.22.4, https://www.qgis.org).

The first sub-region of our experiment is the Baoan District. From the distribution of the diffusion coefficient in Fig. 4, within the time frame of the study, the impact of the spatial neighborhood on the diffusion process of cropland, water, and impervious in Baoan District is still relatively large. During the diffusion process of forest and barren, the influence of neighborhoods on this process is relatively small. Since there are no shrubs or snow in the Baoan District, their diffusion coefficients are 0. At the same time, the areas with large fluctuations in diffusion coefficient are concentrated in the western coastal area of Baoan, which means that the diffusion trend of impervious surfaces in the western coastal area is more obvious during the study period.

From the distribution of the growth coefficients, it is found that the impervious in the Baoan District had obvious self-growth trends while being affected by the neighborhood. Which means that during impervious expansion, the larger the impervious in the spatial neighborhood, the easier it is for the central grid to become new impervious. Similarly, the aforementioned areas of expansion are predominantly clustered within the western coastal region of Baoan.

For the competition coefficient, similarly, cropland and impervious are accompanied by a strong competition coefficient. In addition, the water in Baoan District show a strong competition coefficient, which indicates that, even though the water in Baoan District has an increasing trend from the historical time series, the development of other land cover types often limits the spread of water area.

#### The experiment in Dapeng District

**Figure 5:**
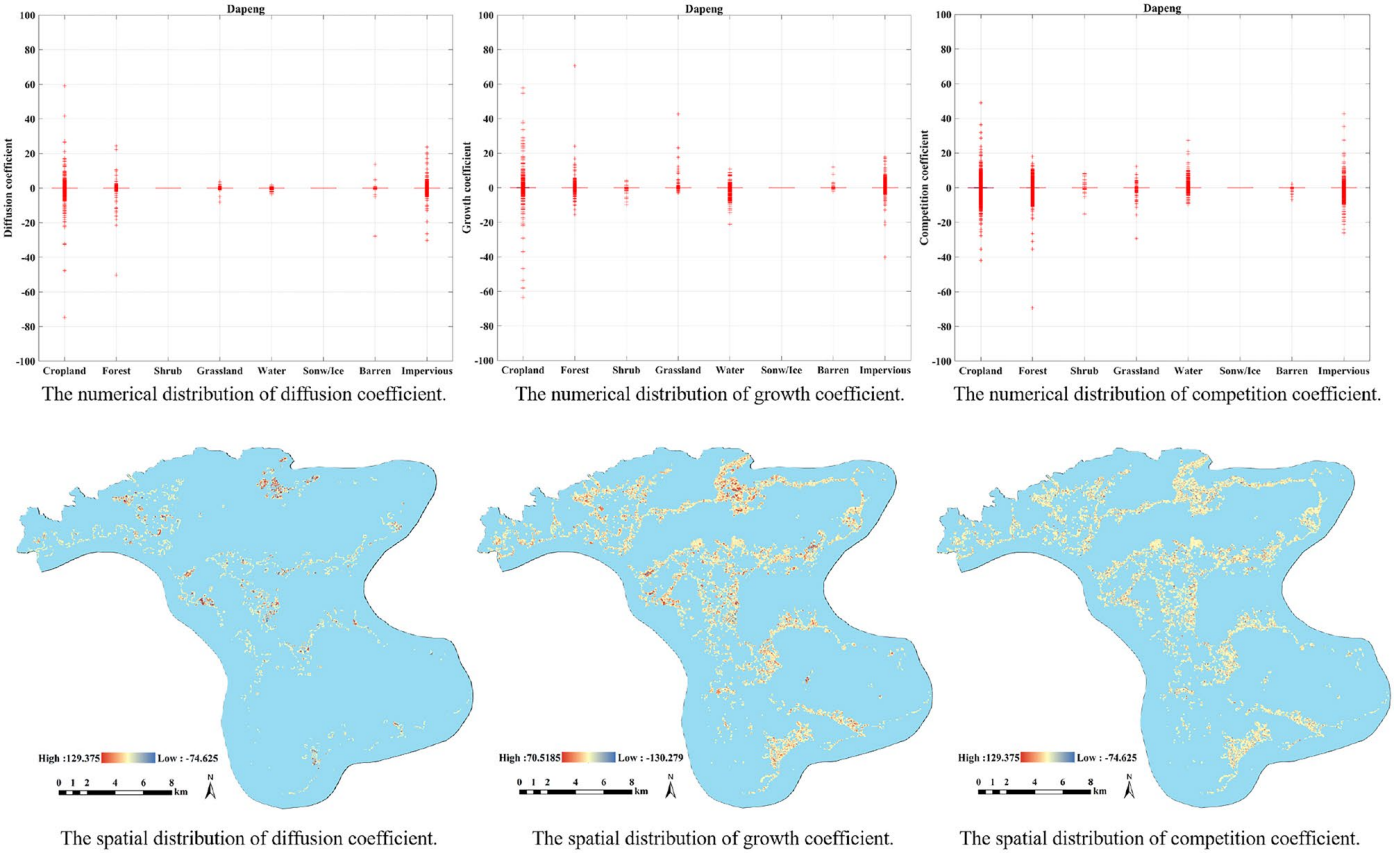
The distribution of different coefficients in Dapeng. The figure was created by QGIS (QGIS Desktop 3.22.4, https://www.qgis.org).

As shown in Fig. 5, Dapeng District is similar to the Baoan District, in that cropland, forest, and impervious are shown to have a strong tendency to be influenced by spatial neighborhood, but the degree of influence is relatively small on the whole. It is reflected in the spatial distribution that only the local values change drastically.

The results of growth coefficient show that in addition to cropland and impervious, forest and water in Dapong also exhibit large growth coefficients. This indicates that the forest and grassland in the Dapeng District have increasingly more possessions during the study period. Correspondingly, in the spatial space, the growth coefficient of the region where the above-mentioned types of changes have occurred is more drastic.

Similar to the Baoan District in the coastal area, apart from farmland and forests, the Dapeng District also have a strong competition coefficient in the category of water, indicating that water is more occupied in the process of urban expansion. This also shows that in the process of land cover change in Dapeng District, water is one of the most impermeable categories in transformation.

#### The experiment in Guangming District

**Figure 6:**
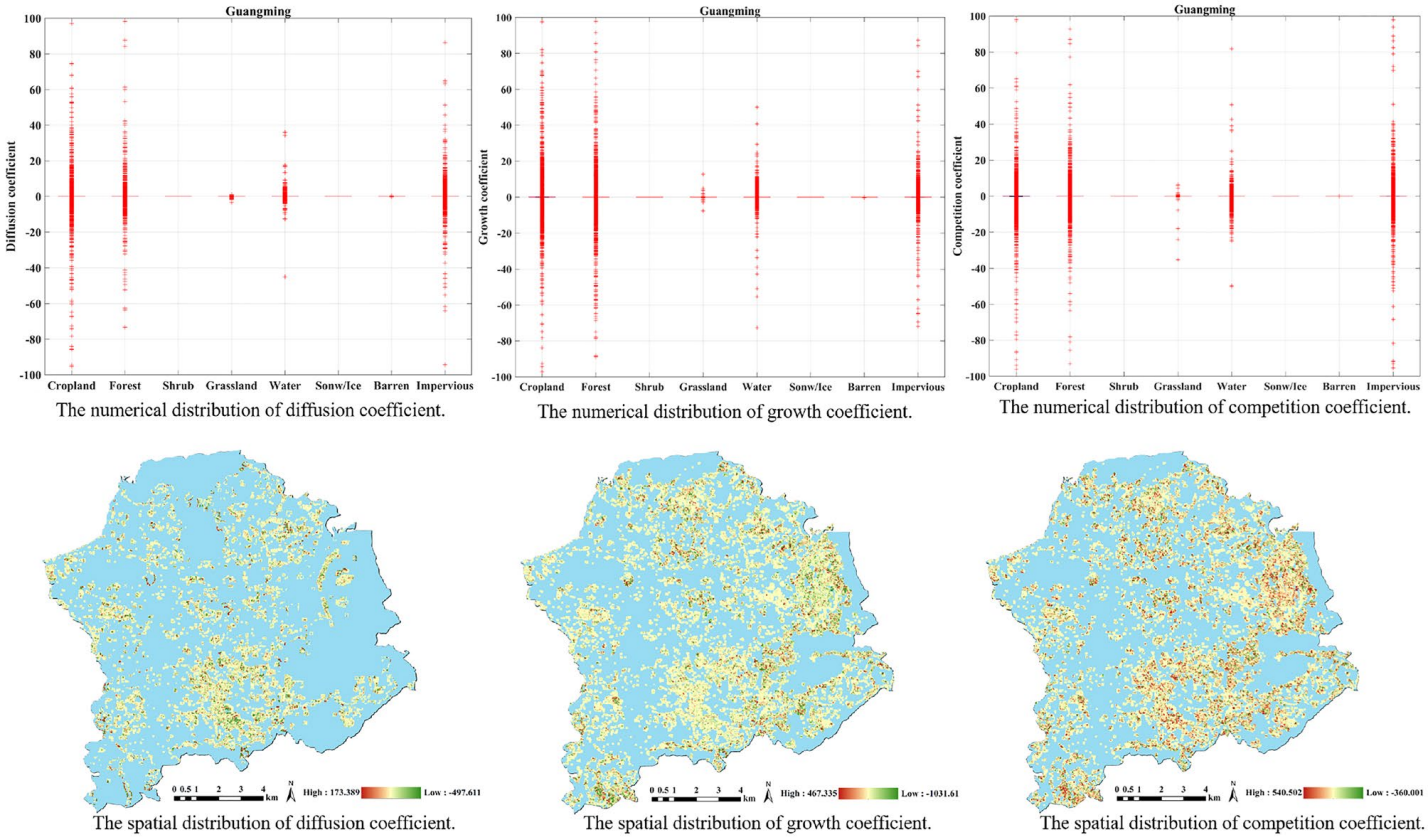
The distribution of different coefficients in Guangming. The figure was created by QGIS (QGIS Desktop 3.22.4, https://www.qgis.org).

From Fig. 6 of the diffusion coefficient, the land cover change in Guangming District is found to be mainly concentrated in the following four types: cropland, forest, water, and impervious. Judging from the distribution of the diffusion coefficients, cropland is more negatively affected by spatial neighbors; that is, the denser the cropland in the surrounding neighborhood, the less favorable it is for outward spread. This may be due to the fact that densely distributed areas of cropland are more likely to be intensively developed as impervious.

Figure 6 exhibits the result of the growth coefficient. From the perspective of growth coefficients, the distribution of the above four types cropland, forest, water and impervious is relatively average, indicating that the impact of historical trends is not easy to evaluate.

The result of the competition coefficient is delineated in Fig. 6. From the perspective of numerical distribution, the distribution of the competition coefficient is similar to that of the growth coefficient, and it is mainly concentrated in the four types of cropland, forest, water and impervious. Similarly, from the perspective of spatial distribution, in areas where the growth coefficients of land cover types change drastically, the corresponding competition coefficients also change sharply.

#### The experiment in Yantian District

**Figure 7:**
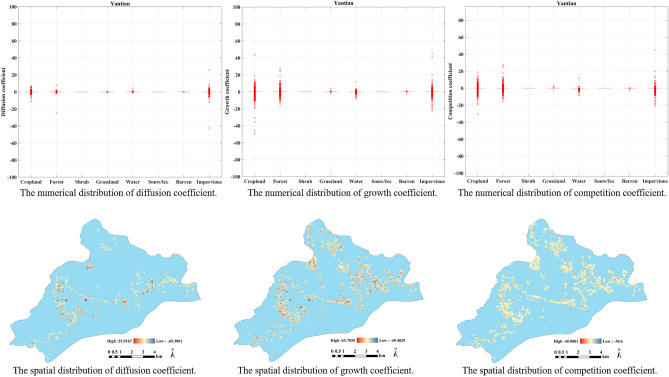
The distribution of different coefficients in Guangming. The figure was created by QGIS (QGIS Desktop 3.22.4, https://www.qgis.org).

As an old city in Shenzhen, Yantian District is not as intense as the other three districts in terms of the diffusion coefficient, growth coefficient, or competition coefficient values. The results of the diffusion coefficients in Fig. 7 presents that the impervious is the most affected by neighbors, followed by cropland and water. Grassland and barren are less susceptible to neighborhood changes.

Similarly, in the results for the growth coefficients, see Fig. 7, the distribution of growth coefficients of various types in the Yantian is on the low side. However, there are occasional outliers in the growth coefficients of cropland, forest and impervious, indicating that these three types are other main factors in the Yantian change. Figure 7 displays the regions where these major changes occurred.

In Fig. 7, the results for the competition coefficient indicates that the three types of cropland, forest and impervious still fluctuated greatly. While the competition coefficient is more spread out in value distribution than the growth coefficient, it is relatively uniform in spatial distribution.

## Discussion

In this paper, we proposed a partial differential equation with cross-diffusion to model the evolutionary process of urban elements. The model explores the development and change process of urban elements and uses the diffusion coefficient to represent the impact on the surrounding neighborhood, and the cross-diffusion coefficient to represent the effects of other elements. The action process of the elements themselves is also reflected by the growth coefficient and the competition coefficient. Through the spatial distribution of these key coefficients, we observed the spatial heterogeneity of the development process of elements at the micro level. Combining the ecological dynamic meaning of these key elements, we were able to obtain a glimpse of the key driving elements or driving forces in the process of element evolution and gain a further understanding of urban elements. At the same time, our model still needs to be improved. The force that urban elements receive during the evolution process is far greater than that of the few items included in the model. Thus, this is still a field worth exploring to examine more action items according to the characteristics of element development.

In the model proposed in this paper, simulating changes in urban elements requires that the impact of the surrounding neighborhood be considered. Therefore, we first needed to define the range of the spatial neighborhood. In this paper, we used the classic Moore neighborhood format, which is also used in the CA model as a spatial neighborhood. In the simulation experiment for population and housing price elements, we regarded housing price as the main factor and population density as the secondary factor, and we evaluated the impact of population density on the development process of housing prices in Shenzhen while considering the developmental characteristics of housing prices themselves. In the experiment with labeled data, specifically land-use data, we regarded the built-up area with the largest newly-added area as the main element, according to the characteristics of the data, and evaluated the impact of other categories on the expansion process of the built-up area while considering the developmental inertia of the built-up area itself.

## Methods

In the process of urban evolution, different elements form specific spatial structures. Not only are elements constantly changing, but they are also constantly affecting other elements neighbored to them. Taking built-up as an example, the marginal diffusion and peripheral diffusion of urban built-up area are dominant in China^41^. In other words, the scope of urban built-up areas has been continuously developed and further expanded in the process of urban improvement^42,43^. Second, the state of the neighborhood around the built-up area will also limit its outward diffusion process. For example, if there are many bodies of water around the built-up area, this will directly affect the direction and speed of the outward spread of the built-up areas. At the same time, the process of out-diffusion of settlement areas will also influence other factors in the city, such as the distribution of population density. After the construction of the built-up area is completed, the area will inevitably become susceptible to settlement, which will lead to changes in the distribution of urban population density.

Taking into account the features of urban development, we made the following assumptions about the process through:

- Constrained by resources and other environmental factors, it is difficult for urban elements to grow indefinitely. The closer a factor reaches the upper limit of capacity, the greater the resistance to sustaining growth. Referring to the competition dynamics model in ecology, we use the competition term to simulate this growth-limited phenomenon of urban elements.
- Under resource scarcity, the diffusion speed of different elements is affected by the density of elements in the surrounding area. When the spatial fraction or density of a given element is greater in a given spatial neighborhood, the subsequent continuous diffusion space becomes smaller, and its own evolution is affected, resulting in a slowdown in the rate of diffusion of elements.
- The growth of one factor is influenced by other factors, and this effect is complex. There may be some mutual attraction, such as in densely built-up areas, where the population density is often high, as are housing prices. Some factors are mutually exclusive; for example, in areas with dense water bodies, the population density will be lower.
- Finally, in a certain spatial neighborhood, this paper refers to the molar neighborhood, the urban elements move along the gradient direction of the concentration difference.Therefore, we assume that the ability of the same element to diffuse along the concentration difference in the spatial neighborhood is the same, and that the diffusion ability of different elements is different.

Combined with the previous work^40^, in this paper, the calculation method for the concentration of urban elements is as follows. For population density data and house price data, we take the value of population density and house price as the corresponding concentration. For land cover data, since each value only represents a category, we take the proportion of land use types within a certain neighborhood as the corresponding concentration value. With this concentration defined, we model the following reaction–diffusion model.

### Construction of a reaction–diffusion model

It is assumed that the continuous change of urban elements can be divided into multiple microstages $\sigma t \, (\sigma t\rightarrow 0)$ within a certain time interval. Theoretically, the change of urban elements is accumulated by n microscopic processes. At the initial time point $t = 0$, the city element does not exist. For the time point $t = \sigma t, 2\sigma t, 3\sigma t,\ldots$, the addition of urban elements in each microprocess occurs in the vacant growth area, which increases the space for the existing distribution of urban elements. If the urban area is regarded as a grid area, then in the expansion process of urban elements, the growth of urban elements in each grid of the urban area obeys the diffusion equation in every microscopic process. That is to say, during the microcosmic change process of urban elements in each urban grid, the change in urban elements obeys a diffusion equation involving time and space. Each item in the equation is constructed on the basis that the diffusion of elements is a spatiotemporal process, so the time rate of change in elements is related to spatial location. At the same time, the elements in each grid not only have an influence on the surrounding neighborhood, but also have a self-growth process and are also related to the distribution of other related elements. Therefore, the cross-diffusion system equation was used for fitting.

Diffusion is a material change process driven by a difference in the concentration of the material. Therefore, when diffusion theory is used to study the diffusion of urban elements, the concentration value of each element must first be determined for the diffusion of each urban element. For label data and non-label data in each spatial grid, the collection methods for concentration values were slightly different. In this study, we referred to a prior work^40^ to obtain values for the solution concentrations for the different types of urban elements.

Next, we divided the spatial region of the entire city into a regular grid. In this case, we let $x\in \Omega$ represent the spatial grid, and $\Omega$ was the study area. $t > 0$ represents the moment when urban element data were sampled, and $\{i=1,2,\ldots ,n\}$ represents different urban element types. Then, $U_i(x,t)$ represents the concentration value of element *i* located on spatial grid *x* at time *t*.

Finally, we considered the influence of urban elements on the evolutionary process. The diffusion process of urban elements is first influenced by the change of the same element in the spatial neighborhood and also by other related elements. At the same time, the growth of urban elements is always constrained by various factors, making unlimited growth difficult. In summary, our diffusion model needed to reflect the impact of the above three functional relationships on the extent of urban element development.

In accordance with existing work^44,45^, urban elements were divided into grids with equal spatial size according to the scope of time and space. The following reaction–diffusion equations were constructed to determine the evolution process of the elements in each of the grids. We used reaction–diffusion equations with cross-diffusion terms to describe the spatiotemporal evolution process of elements in the city. The equations had the following form:

1 $$\begin{aligned} \frac{\partial U_i(x,t)}{\partial t}=a_i(x)\Delta U_i+U_i(x,t)(r_i(x)-K_i(x)U_i(x,t))+\sum _{j\ne i}^nb_j(x)\Delta U_j(x,t). \end{aligned}$$

Among these variables, $U_i(x,t)$ and $U_j(x,t)$ show the state of the element in the grid of *x* at time *t* for the element $\{i,j = 1,2,3,\ldots ,n\}$. The $a_i(x)$ represents the influence of elements on the surrounding neighborhood during the evolution process and the promotion or restriction of the difference from the surrounding neighborhood to its own development. For example, when the built-up area is surrounded by bare land, this means that the area may be under development, and the scope of the built-up area will continue to expand.

2 $$\begin{aligned} \Delta =\frac{\partial ^2 U_i(x,t)}{\partial x^2} \end{aligned}$$

is Laplace operator, which represents the diffusion of urban elements in space.

The $r_i(x)$ refers to the growth process of the element itself, and the driving force of this process may come from the historical development trend or its own development (such as population growth). $K_i(x)$ represents the strength of the internal competition of elements. This means that when the concentration of elements is too high, the intensity of competition between neighbors will become larger.

The cross-diffusion term, $b_j(x)$, describes the interaction between different elements $U_j(x,t)$. When $b_j(x)$ was positive, it indicated that the current element was flowing in the direction of the low density of the influencing element. A negative cross-diffusion term coefficient indicates that the current element flowed from the low-density direction of the associated element to the high-density direction.

Since our model (1) aim to form the evolution of urban elements, the model has a non-negative initial value condition:

3 $$\begin{aligned} U_i(x,0)\geqslant 0. \end{aligned}$$

Define the boundary of the study area is $L\in \Omega$. We also assume that no external input is imposed from outside. Hence zero flux boundary conditions

4 $$\begin{aligned} \frac{\partial U}{\partial x}\vert _{L}=0 \end{aligned}$$

is assumed.

### The solution of the model

Since the evolution data of the actual urban elements are all discrete, in order to solve the problem, we need to discretize the above diffusion equation (1). Then we get:

5 $$\begin{aligned} \begin{aligned} \frac{U_i(x,t+T)-U_i(x,t)}{T}=&\,a_i(x)\left[ \frac{U_i(x+X,t)-2U_i(x,t)+U_i(x-X,t)}{X^2}\right] \\&+r_i(x)U_i(x,t)\left[ 1-\frac{U_i(x,t)}{K_i(x)}\right] \\&+\sum _{j\ne i}^nb_j(x)\frac{U_j(x+X,t)-2U_j(x,t)+U_j(x-X,t)}{X^2}, \end{aligned} \end{aligned}$$

where *T* is the time interval, *X* is the space interval.

When X and T are given, after rearrangement, the following iterative equation is obtained:

6 $$\begin{aligned} \begin{aligned} U_i(x,t+T)=&\frac{a_i(x)T}{X^2}[U_i(x+X,t)-2U_i(x,t)+U_i(x-X,t)]+U_i(x,t)\\&+\frac{T}{X^2}\sum _{j\ne i}^nb_j(x)[U_j(x+X,t)-2U_j(x,t)+U_j(x-X,t)]\\&+Tr_i(x)U_i(x,t)\left[ 1-\frac{U_i(x,t)}{K_i(x)}\right] . \end{aligned} \end{aligned}$$

According to the iterative formula and the evolutionary data of the urban elements, we use a series of optimization algorithms to solve the parameters in the model. Due to the fast convergence speed of the particle swarm optimization (PSO) algorithm, the algorithm did not have many parameters that needed to be adjusted. For this paper, we used the PSO to identify the parameters in our model (6).

## Materials

In this section, we describe the basic settings of the research area, and then we provide a detailed account of the source of data acquisition and the attributes of the data. In this article, we use the population density, housing price and land cover data of Shenzhen, China. We further demonstrate the preprocessing of the data for our experimental procedure. Finally, we illustrate some basic settings of the relevant parameters during our experiments.

### Study area

**Figure 8:**
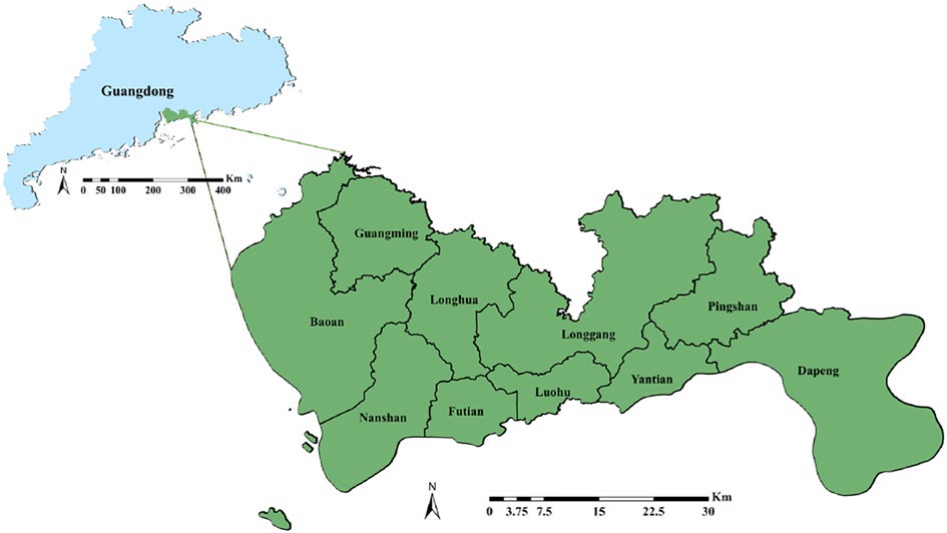
The geographical location and administrative division of Shenzhen. The figure was created by QGIS (QGIS Desktop 3.22.4, https://www.qgis.org).

Shenzhen is a sub-provincial city in Guangdong Province and a special economic zone in China. There are nine administrative districts and one new district under the jurisdiction of the city. As shown in Fig. 8, Shenzhen is located in the south of Guangdong Province, on the east bank of the Pearl River Estuary, between $113^\circ \, 43'$ E to $114^\circ \, 38'$ E and $22^\circ \, 24'$ N to $22^\circ \, 52'$ N. Shenzhen is one of China’s central economic cities. Its total economic output has long been ranked fourth among the cities in mainland China, marking it as one of mainland China’s cities with the best economic benefits. Since 2012, Shenzhen’s year-end resident population has increased from 11.9585 million to 17.6816 million (from the Shenzhen Municipal Bureau of Statistics). The gross regional product also increased from 349.442 billion in the first quarter of 2015 to 573.403 billion in the first quarter of 2019.

### Data and processing

In the study of urban elements, land-use change has attracted the most attention. Urban land change focuses on the simulation and prediction of the change trend, and even more so on the driving force of land change. Therefore, different models can be used, such as CA, the multi-agent model, and machine learning. In addition, more than half of the population lives in cities, and a shift in population distribution is one of the most significant changes in urbanization. House price, in certain areas of economics, has been evaluated with many statistical methods over the last 30 years, some of them focusing on spatial aspects.

Because of this, in this paper, we mainly used data on land cover, population density, and house price in Shenzhen. The land cover data for Shenzhen came from the annual China Land Cover Dataset (CLCD) dataset^46^, which was published by the Wuhan University team. The CLCD dataset is an annual China map coverage dataset based on all available Landsat data on Google Earth Engine (GEE) combined with random forest classifiers to obtain classification results. It has a spatial resolution of 30 m and contains year-by-year land cover information for China from 1985 to 2021. Based on 5463 samples of visual interpretation, CLCD has an overall accuracy of 80%. Moreover, the CLCD dataset showed good consistency with global forest change, global surface water, and impermeable surface time-series datasets.

For population data, we used the top-down population density data provided on the WorldPop website. Existing studies have shown that in Guangdong Province, China, WorldPop has spatial distribution consistency with GPW v4 and two Chinese kilometer grid population distribution datasets. Compared with the sixth national census data as the true value, the overall accuracy of the WorldPop dataset is higher. The highest spatial resolution of the population density data provided on WorldPop is 100 m. To be consistent with the resolution of the land cover data, we resampled it to 30 m and cropped the data of Shenzhen. Finally, our house price data came from the data on Fangtianxia and Anjuke, which were converted into raster data with a spatial resolution of 30 m. Three kinds of data with the same coordinate system, spatial range, and temporal and spatial resolution were thus obtained. The time range of the three data types was from 2011 to 2019.

### Experiment parameter setting

In the model proposed in this paper, simulating changes in urban elements requires that the impact of the surrounding neighborhood be considered. Therefore, we first needed to define the range of the spatial neighborhood. The currently used spatial neighborhood types include 4-neighborhood, 8-neighborhood, and 25-neighborhood formats. In this paper, we used the classic 8-neighborhood format, which is also used in the CA model as a spatial neighborhood. In the simulation experiment for population and housing price elements, we regarded housing price as the main factor and population density as the secondary factor, and we evaluated the impact of population density on the development process of housing prices in Shenzhen while considering the developmental characteristics of housing prices themselves. In the experiment with labeled data, specifically land-use data, we regarded the built-up area with the largest newly-added area as the main element, according to the characteristics of the data, and evaluated the impact of other categories on the expansion process of the built-up area while considering the developmental inertia of the built-up area itself.

## Data Availability

The CLCD dataset introduced in this article is freely available at https://doi.org/10.5281/zenodo.4417810. The population density data of China can been download at https://www.worldpop.org. The house price data can be found at https://github.com/minjincug/House_price_data. The administrative division data of Guangdong Province and Shenzhen City are downloaded from https://guangdong.tianditu.gov.cn.
